# Assessment of Bonding Effectiveness of Adhesive Materials to Tooth Structure using Bond Strength Test Methods: A Review of Literature

**DOI:** 10.2174/1745017901814010664

**Published:** 2018-09-28

**Authors:** Aminah M. El Mourad

**Affiliations:** Department of Restorative Dental Sciences, King Saud University, Riyadh, Saudi Arabia

**Keywords:** Adhesive, Bond strength, Shear, Tensile, Push-out, Micro-shear, Micro-tensile, Micro- push-out

## Abstract

**Background:**

The rapid developments in the field of adhesive dental materials have led to improvements in many aspects of clinical dentistry. Adhesive bond strength plays an important role in determining the clinical performance and longevity of dental restorations. Nevertheless, bond strength tests have never been well-standardized, although a number of important recommendations have been made.

**Objective:**

The aim of this paper is to critically review the validity of different bond strength testing methods for assessment of bonding effectiveness of adhesive materials to tooth structure and discuss factors that may affect bond strength measurement.

**Data Collection:**

Relevant literature published between 1983 and 2018 was collected and reviewed from the PubMed database and Google scholar resources.

**Review Results:**

Results of the current bond testing methods should be used to compare materials tested under the same laboratory settings, but they shouldn’t be used to make direct inferences on their clinical behaviour. Shear and micro-shear tests, result in non-uniform stress distribution, stress concentration at the substrate area, and predominantly tensile stresses rather than shear stresses. Micro-tensile bond tests provide many advantages over the shear tests, although these methods are technique sensitive and labour intensive.

**Conclusion:**

Bond strength testing methods should be well-standardized, but there are many factors that cannot be fully controlled which leads to variation and misinterpretation of the data about the bonding abilities of adhesives.

**Clinical Significance:**

New adhesive materials should be subjected to a combination of testing protocols to properly assess their bonding effectiveness.

## 1 BACKGROUND

Dental adhesive technology has experienced rapid developments in recent years. Manufacturers continue to introduce new adhesive systems with claims about the ease of use, enhancement in adhesive composition, and improvement in adhesives’ bond strength to the tooth structure. Bond strength measurement tests are used worldwide to substantiate these claims and to evaluate bonding effectiveness of different adhesive systems to the tooth structure. Bond strength testing of adhesive systems is considered a reliable predictor of the longevity of dental restorations. Bond strength is defined as the initial mechanical load to fracture divided by the simple, geometrically defined, cross-sectional area of the bond. Nevertheless, bond strength tests to predict the clinical performance of dental adhesives have never been well-standardized, although a number of important recommendations have been made [1]. Bond strength can be measured following different types of testing methods. There are many factors that can affect the resultant bond strength values, although the validity of these test methods is questionable. Thus, this paper aims to critically review the validity of different bond strength testing methods to assess adhesives’ bonding effectiveness to the tooth structure and discuss related factors affecting the bond strength measurement.

## 2 DATA COLLECTION

Inclusion criteria of this literature review included relevant articles published between 1983 and 2018 which were collected and reviewed from the PubMed database and Google scholar resources.

## 3 REVIEW RESULTS

This literature review provides fundamental information about the validity of different bond strength testing methods and factors that affect bond strength measurement.

## 4 TYPES OF BOND STRENGTH TESTING METHODS

**Fig. (1):**
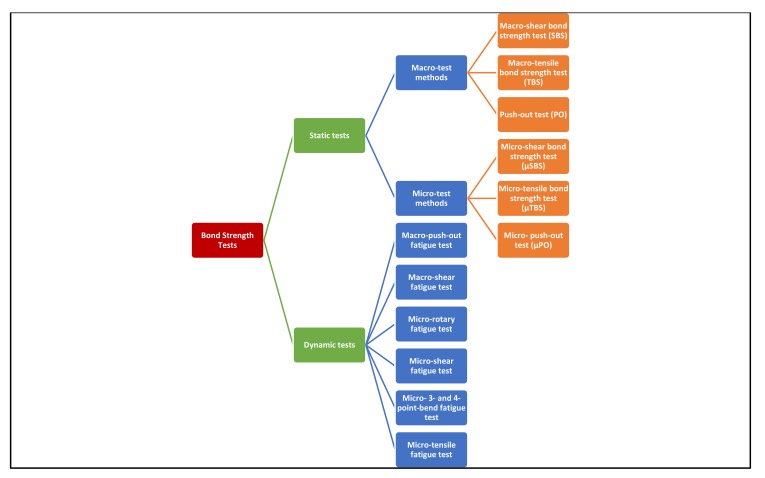
A summary of laboratory bond strength testing methods.

Bond strength can be measured by either laboratory methods or by evaluation of bond durability and clinical performance. Laboratory bond strength testing methods are divided into static and dynamic tests (Fig. **1**). In static tests, load is applied while the specimen is held fixed, whereas in dynamic tests, the specimen is in a dynamic state. Static tests are divided into macro and micro tests according to the size of the bond area. The macro-bond strength test, with a bond area larger than 3 mm^2^, can be measured using ‘shear’, ‘tensile’, or ‘push-out’ protocol, while in the micro-bond test, the bond area tested is much smaller, about 1 mm^2^ or less [2].

### 4.1 Static Tests

#### 4.1.1 Macro-Test Methods

They are divided into shear, tensile, and push-out test methods.

##### 4.1.1.1 Macro-Shear Bond Strength (SBS) Test

**Fig. (2):**
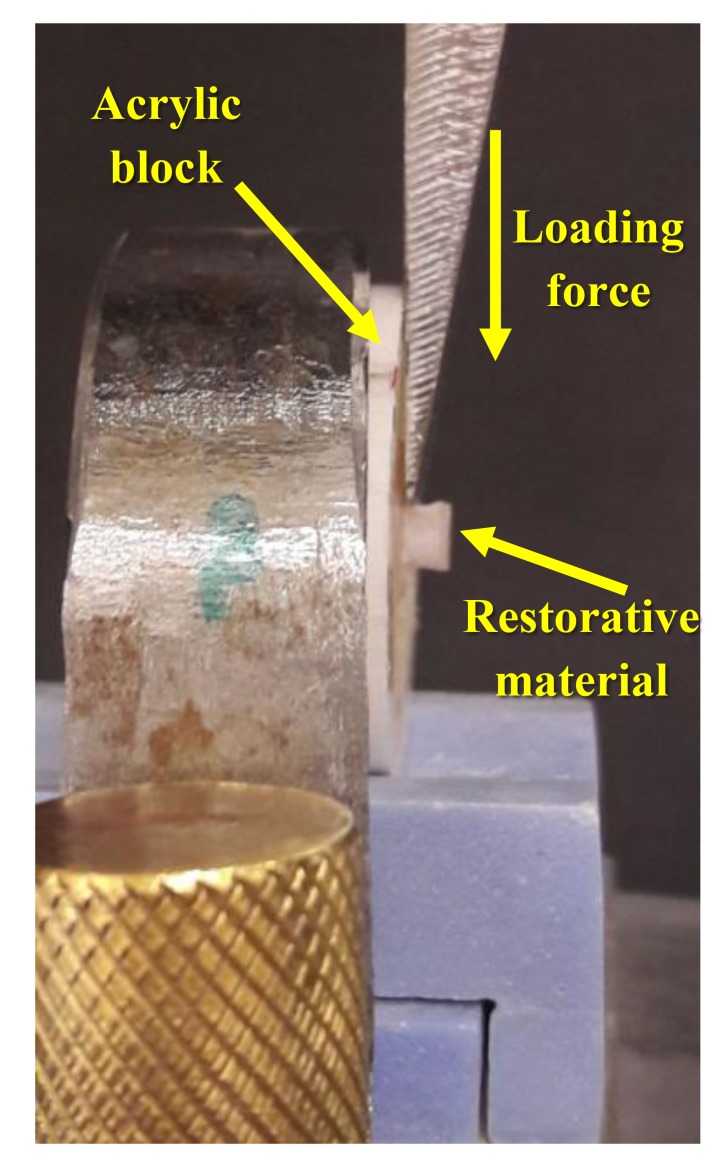
Shear bond strength test set-up.

The Macro Shear Bond Strength (SBS) is the most commonly used test to screen new adhesive formulations according to their bonding effectiveness [3]. This test method was first described by Bowen in 1965 [4]. The SBS is defined as the maximum stress that a material can withstand before failure in a shear mode of loading. In a shear bond test, two materials are connected by an adhesive agent and loaded in shear until fracture occurs (Fig. **2**). The SBS test gains its high popularity in companies and research institutes since no further specimen processing is needed after the bonding procedure; thus, it is the easiest and fastest method [5]. However, cohesive failures in the substrate were frequently observed with new adhesives that show improved bond strengths, which affected the validity of obtained measurements with this test method. The explanation for this fact was that stresses were mostly concentrated in the tooth substrate, thus causing its premature failure before to the interface itself [6-10].

##### 4.1.1.2 Macro-Tensile Bond Strength (TBS) Test

**Fig. (3):**
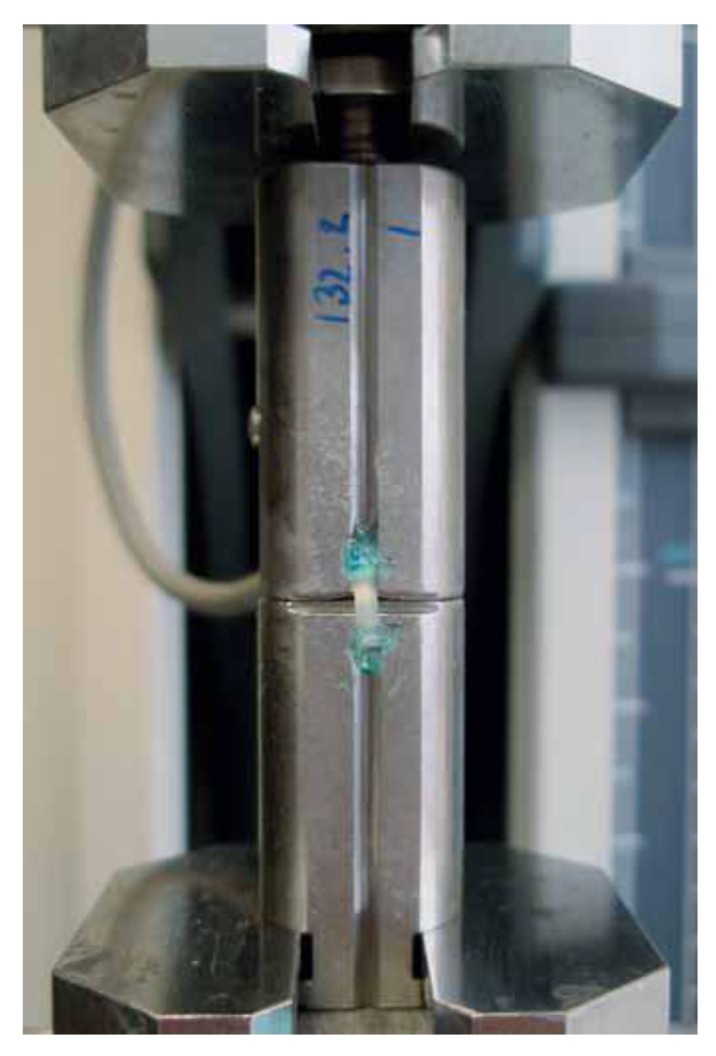
The ends of a test specimen attached for tensile bond strength test.

The Macro Tensile Bond Strength test (TBS) is considerably less commonly used test. It is used to indicate the bond strength of cement to other hard materials such as ceramics and metal alloys [11, 12]. Because the distribution of stress is considered much more uniformly in TBS tests than in shear tests, TBS provides a more accurate estimate of the stress level that initiates bond breaking [13]. In a TBS test, the load is applied on either side of the test specimen [14]. A perpendicular alignment of the bonded interface of the specimen to the loading axis is very important with tensile tests; otherwise, bending stresses will develop. Thus, the test specimen should be attached to the mechanical testing machines by active or passive gripping approaches (Fig. **3**).

##### 4.1.1.3 Push-Out (PO) Test

**Fig. (4):**
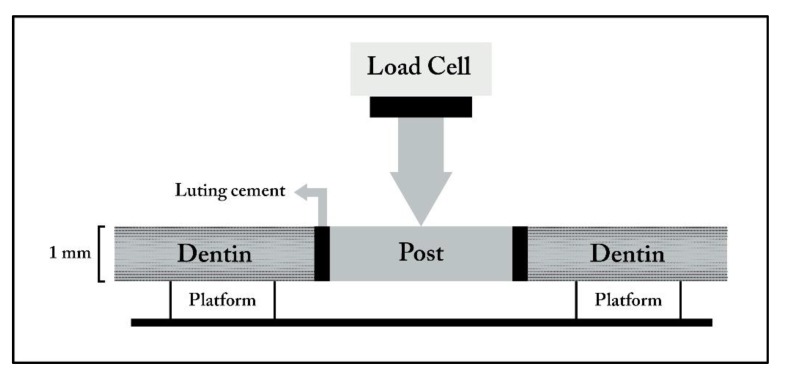
Schematic representation of the push-out bond strength test.

A Push-Out (PO) approach has been used to dynamically test the fatigue resistance of adhesive-dentin bonds. This method is very useful in testing the adhesion of root canal sealers and retention of posts luted in root canals [15]. The PO test is based on generating shear stress at the interface between dentin and cement, as well as between post and cement [16]. When the PO test is used to test bond strength of adhesives to dentin, a 1 to 2 mm thick dentin slice is punched to produce a tapered cylindrical hole. The internal surface of the hole is treated with an adhesive, and the hole is loaded with composite. Then, the composite cylinder is pushed through the dentin from the smaller diameter side, and the bond strength is calculated by dividing the extrusion force by the lateral area of the tapered cylinder (Fig. **4**). This test provides more accurate information on the effect of different adhesives on bond strength compared with the conventional shear bond test because it includes extraction of the curing composite. As a result, failure occurs parallel to the post-cement-dentin interface, which simulates the clinical condition more closely [17]. However, PO has never been accepted as a universal bond strength test method, probably because of the laborious steps in specimen preparation and time-consuming methodology.

#### 4.1.2 Micro-Test Methods

They are divided into micro-shear, micro-tensile, and micro-push-out test methods.

##### 4.1.2.1 Micro-Shear Bond Strength (µSBS) Test

The Micro-Shear Bond Strength (µSBS) test was introduced in 2002 [18]. and allows testing of small tooth areas. The test results in a depth profiling of different substrates and preparation of multiple specimens from a same tooth as in the case of micro-tensile tests. However, without the involvement of sectioning or trimming procedures which by themselves may induce early microcracking within the specimen [19, 20].

The general findings based on finite element analysis and failure mode analysis of SBS testing also holds true for µSBS testing, and include: 1) tensile stresses produced by the bending moment at load application being responsible for fracture initiation, 2) highly non-uniform stress distribution concentrated in the substrate even more pronounced with µSBS test compared to SBS test, and 3) a nominally measured bond strength that severely underestimates the true stress the specimen resisted at fracture [21]. In a finite element analysis study by Placido *et al*., [22], it was concluded that µSBS testing represented the shear bond strength less effectively than in the conventional SBS test. This difference was attributed to the relatively thicker adhesive layer and farther load application from the adhesive joint.

The µSBS test methods are used to test substrates with properties such as glass ionomers or enamel that make them particularly prone to the specimen preparation effects and testing conditions of micro-tensile bond testing [19, 23].

##### 4.1.2.2 Micro-tensile Bond Strength (µTBS) Test

**Fig. (5):**
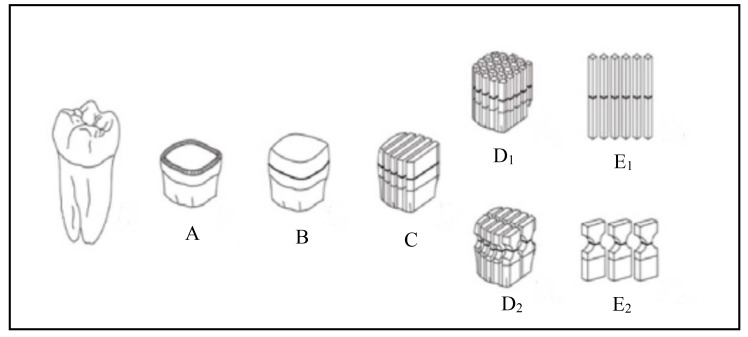
Schematic illustration of the acquisition of specimens for micro-tensile bond strength test. (**A**) Removal of occlusal surface of the tooth to expose dentin; (**B**) Bonding procedure at the tooth surface and construction of composite resin blocks; (**C**) The specimen is vertically sectioned into multiple serial sections; (**D_1_, E_1_**) Non-trimmed specimens (stick-shaped); (**D_2_, E_2_**) Trimmed specimens.

This test method was first developed by Sano **et al**., [24], in 1994, it involves bonding adhesive resins to the entire flat occlusal surface of teeth, which is then covered with a resin composite. After curing and storage in water, the specimen is vertically sectioned into multiple serial sections with a slow-speed diamond saw (Fig. **5**). The resulting slabs are composed of an upper half of resin composite and a lower half of dentin, using an ultrafine diamond bur; the cross-sectioned area at the bonded interface should be reduced to form an hour-glass shape to ensure maximum stress development at that region [25].

Because the µTBS method requires further specimen processing after the bonding procedure, this makes the test more difficult, technique sensitive, and results in dehydration of the smaller specimens [26]. In addition, the drawbacks of µTBS method include difficulty in measuring bond strengths lower than 5 MPa, difficulty in fabricating specimens with consistent geometry, and easy damage of specimens. However, the advantages of this method are the better control of regional differences, the better economic use of teeth, and the better stress distribution at the true interface. The greatest advantage of the technique is that one can obtain exclusively adhesive bond failures of materials if the bonded surface area is about 1 mm^2^ because of the better stress distribution at the true interface [27,28].

Several micro-specimen preparation procedures are usually used to produce different specimen shapes such as ‘trimmed’ or ‘non-trimmed’ micro-specimens [29]. Stick-shaped, non-trimmed specimens are preferred for enamel specimens as they can be prepared in a less destructive, easier, and more accurate way. Trimmed micro-specimens at the interface to hourglass-shaped specimens allows better concentration of stress at the interface [30]. However, if this trimming is not carefully performed, mechanical stress and attrition will be induced, leading to cracks in tooth structure and causing the interface to fail prematurely at lower bond strength [30-32]. In addition, such micro-specimen trimming using dental handpieces largely depends on the skills of the operator; therefore, it is highly recommended to use the semi-automatic trimming of micro-specimens using Micro Specimen Former (University of Iowa, Iowa City, IA, USA) to trim specimens in a standardized way. Moreover, there are other factors that might affect the µTBS results, including specimen-jig attachment, specimen-loading speed, and specimen alignment. Therefore, these procedures should be standardized during the test setup [32].

One issue frequently seen with µTBS test is the pre-testing failure [33]. In general, adhesives with high µTBS do not suffer from these pre-testing failures. To avoid the occurrence of such failures, micro-specimen preparation should be atraumatic and special measures should be taken, such as the use of alginate and gypsum to fill up the space between the slabs during the cutting procedure.

##### 4.1.2.3 Micro- Push-out (µPO) Test

The Micro-Push-Out (µPO) test is employed to measure the bond strength of luted fibre posts. It is a modification of the PO test, and it involves disks of a radicular dentin specimen with thickness less than or equal to 1 mm^2^ [34, 35]. Castellan **et al**., [36] reported that a µPO approach and µTBS resulted in higher values than traditional pull-out and push-out methods. However, this µPO method needs further investigation.

### 4.2 Dynamic Tests (Fatigue Tests)

A static bond strength test is regarded as clinically less relevant since such sudden loading of the adhesive-tooth bond clinically never occurs. Therefore, dynamic fatigue testing using cyclic subcritical loading is usually claimed to better predict the clinical effectiveness of dental adhesives. Only a few number of fatigue tests have been performed in recent years because they are more labour intensive and time-consuming than static bond strength tests. In addition, these fatigue tests have largely been applied to dentin, since bonding to enamel being much more difficult to assess in fatigue [37].

Many fatigue tests have been reported in the literature: **1**) Macro-push-out fatigue test [38], **2**) Macro-shear fatigue test [39], **3**) Micro-rotary fatigue test [40], **4**) Micro-shear fatigue test [22], **5**) Micro-3 and 4-point-bend fatigue test, and **6**) Micro-tensile fatigue test [41]. Among them, 3-point and 4-point bending fatigue tests have evolved to be the most common. In 3-point bending test, a non-uniform stress under the loading piston is created, whereas in the 4-point test, the stress field between the support rolls is uniform, which can lead to different flexural strength findings and less premature failures. The 3-point bending test can be used where the tested material is homogeneous, while 4-point bending test tends to be the best choice if the material is not homogeneous.

## 5 BOND DURABILITY ASSESSMENT

Clinical evaluation of bonding durability under complex oral environment is highly recommended; however, *in vitro* testing is required to explicate the specific factors that cause bond deterioration over time. To minimize the differences between *in vivo* and *in vitro* conditions, challenging the adhesive interface under more clinically relevant circumstances or upon aging of the specimens should be conducted. It was claimed that laboratory bond strength testing cannot predict clinical effectiveness of adhesives due to the wide variation in bond strength values recorded for one specific adhesive between different research institutes worldwide [42]. The main reason for these inconsistent bond strength data is the lack of a standard bond strength testing protocol [42]. The immediate bonding effectiveness of contemporary adhesives is usually found to be quite favourable, regardless of the approach used. In the long term, the bonding effectiveness of some adhesives drops dramatically, whereas the bond strengths of other adhesives are more stable. In many bond strength studies, some kind of ‘aging’ factor is added to the study methodology [2]. Water storage and thermocycling are the most common aging methods, but other methods such as mechanical loading, pulpal pressure usage, and degradation by enzymes have been utilized in the literature [43, 44]. It was reported that within about 3 months of water storage, all classes of adhesives demonstrated an evidence of degradation that simulates *in vivo* aging [45-47].

## 6 FACTORS THAT AFFECT BOND STRENGTH MEASUREMENT

Factors that affect bond strength measurement are shown in Fig. (**6**).

### 6.1 Substrate Related Factors

#### a- Source and type of substrate

Many investigators have used bovine teeth as substitutes for human teeth because of the difficulty in collecting intact extracted human teeth for laboratory studies [48, 49]. However, bovine coronal dentin has larger dentinal tubules than in human dentin. Similar bond morphology to human dentin is achieved only when superficial dentinal layers are used. Bovine root dentin and dentin nearer to the pulp produce markedly different results; [50] therefore, the use of human teeth is preferable to obtain valid and reliable results [4].

Many types of teeth were used to study bond strengths. One study reported significant differences between the bond strength values of teeth in the upper and lower arches and found that enamel shear bond strength was significantly affected by both tooth type and adhesive system [51].

#### b-Condition of substrate

Human third molars are often collected for bond testing; however, they are much more permeable and wetter than erupted teeth. In addition, most dentin bonding is done to previously restored teeth, carious teeth, or abraded lesions, which contain sclerotic dentin [4]. Sclerotic dentin is less etchable because it contains tubules that are generally occluded by mineral crystals [52]. Thus, the bond strengths to such dentin are believed to be lower than those of unerupted third molars.

#### c-Dentin depth

Pashley **et al**., [53] examined the shear bond strengths to superficial, intermediate, and deep dentin with four dentin bonding systems and confirmed higher bond strengths in superficial dentin and progressively lower bond strengths in deep dentin. This was attributed either to differences in chemical composition of tested adhesives or regional differences in wetness (dentin permeability) [25]. Shear bond strength results were known to decrease with increased dentin depth and permeability [54]. In addition, a general reduction of the sensitivity of shear bond strengths to dentin depth is reported along with the use of more hydrophilic adhesive systems [55].

#### d-Enamel prisms and dentinal tubules orientation

The variation in enamel bonding locations might have an effect on the bonding ability of many adhesive systems because of the enamel’s structural anisotropy and its prismatic, rod-like apatitic morphology. Many studies showed that the tensile strength of enamel is dependent on the prismatic orientation [56]. Ikeda **et al**., [57] demonstrated lower µTBS in specimens stressed perpendicular to the prism long axis than in specimens stressed parallel to the prism axis. Shimada and Tagami [58] showed that the effect of the orientation of the prismatic structure of the enamel on the bond strength results is adhesive dependent.

The direction of dentin tubules was reported to be an important factor that affects the bond strength of contemporary adhesive materials. Cehreli and Akça [59] found that samples bonded parallel to the dentin tubules showed the highest µTBS, followed by the oblique and perpendicular groups. However, another study indicated that bond strength results were not influenced by tubule orientation [60].

#### e-Pulpal pressure

Pulpal pressure is used in some bond strength tests to simulate *in vivo* conditions. Numerous studies reported that pulpal pressure leads to a decrease of μTBS in many bonding systems [61, 62]. Alexandre **et al**., [63] indicated that simulated pulpal pressure presented different effects on the long-term adhesive performance of the resin cements and the effect usually depends on the type of tested adhesive.

#### f-Storage media and duration

Many solutions were used as aging media including distilled water, artificial saliva, and sodium hypochlorite (NaOCl). One study reported that six months of water storage did not cause any change in μTBS [64]. However, another study indicated that the resin-dentin bond was subjected to water degradation after four years of water storage [65]. Lee **et al**., [66] concluded that storage in NaOCl resulted in significantly lower bond strength than in distilled water, sodium chloride, chloramine-T, glutaraldehyde, and formalin. This was attributed to deterioration of dentin mechanical properties by NaOCl [67].

The effect of storage duration on bond strength after extraction was shown to be controversial. Several studies reported that storage duration had no significant effect on the shear bond strength of resin to dentin [68]. In contrast, another study involving storage of teeth in formalin and thymol solutions for 6 months did have a negative influence on bond strength because of dissolution of the smear layer [69].

### 6.2 Specimen Related Factors

#### a-Specimen size

The size of the specimen affects the size of the bonding area. The effect of the bonding area on the SBS and TBS was evaluated in two different studies. Both studies concluded that bond strength decreased with increased surface area [70-72].

The preparation of the micro-specimens is considered to be more technique sensitive. Many studies reported that micro-bond strength was also reduced when the bonding area was increased [6, 72].

#### b-Specimen shape

The effect of the specimen shape on the micro-bond strength was previously discussed in the μTBS section.

#### c-Thermal cycling and mechanical loading

Thermal cycling and mechanical loading have been included in many studies to simulate the clinical situation. The thermal cycles in the oral environment can lead to deteriorating stresses between the tooth substrate and restorative material by producing expansion and contraction stresses [73]. Many studies found that thermocycling reduced the bond strength to enamel and dentin depending on the type of adhesive [73, 74]. Burke **et al**., [75] reviewed the methods used in bond testing in 102 published investigations and found that 82% of the papers did not provide information on whether the specimens had undergone thermal cycling. If stated, it was reported that the values ranged from 100 cycles to 2500 cycles with a mean range of 5-55˚C. The majority of those studies reporting performance of thermocycling indicated that it had no significant effect on the bond strength values. It is recommended to perform thermocycling at a frequency of 500 cycles by ISO/TS specification for reliable results [76]. If it exceeds this frequency, studies have shown decrease of bond strength [74,77].

In clinical conditions, teeth are constantly encountering stresses during mastication and parafunctional habits. As previously mentioned, fatigue tests are more recommended for better clinical relevance [78]. During a fatigue test, a metallic plunger is connected to a cyclic loading machine and placed in contact with the restoration, and a fixed amount of cyclic axial load is distributed at a frequency of 2-3 cycles/s [79]. Many studies have demonstrated that masticatory loadings could result in degradation of dentin bonding interface [80, 81].

#### d-Elastic modulus of the resin composite

Mechanical properties of the composite can affect the bond strength test results. The effect of the composite modulus of elasticity on stress distribution at the bonded interface is usually assessed by finite element analysis. It was found that the use of stiffer composites may significantly increase bond strength values [82]. Researchers concluded that stress concentration at the bonded interface decreased as the composite modulus increased [13]. The influence of the composite elastic modulus on bond strength was found to be adhesive dependent [82].

#### e-Operator skill

Operator skill in material handling and test apparatus usage appears to play an important role in determining the outcome of bond strength testing, the efficiency of which can be improved with repeated use of bond strength tests and materials [83].

### 6.3 Test Mechanics Related Factors

#### a- Configuration of the loading device

**Fig. (7):**
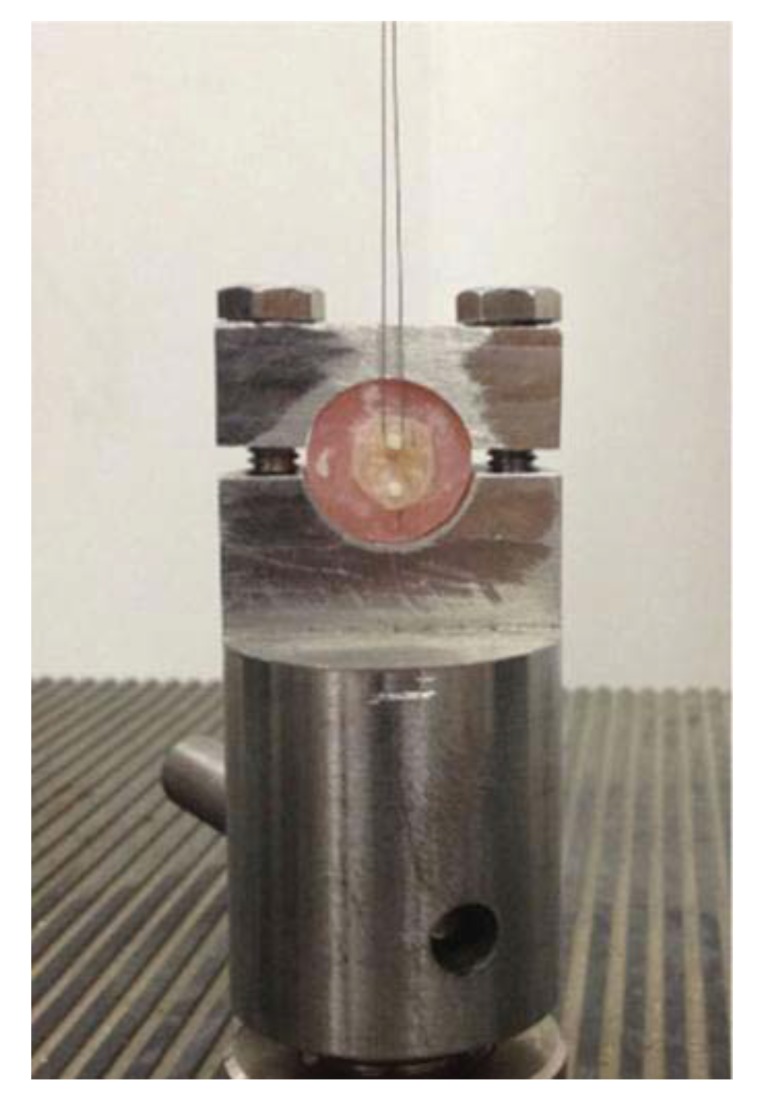
Micro-shear bond strength test using wire-loop loading.

The configuration of the loading device influences the stress distribution at the bonded interface, affecting the bond strength. The higher the stress concentration in the load application area, the lower is the bond strength. Studies concluded that the use of a knife-edge chisel results in lower bond strength values than that of the wire loop, wherein the load is distributed over a larger area [84]. Moreover, it was found that the loading with stainless steel tape allowed more uniform stress distribution at the bond interface and was a more reliable way to evaluate the bond strength. Many studies still use the wire-loop to apply the shear load (Fig. **7**). This shows the importance of evaluating the other loading devices [84].

#### b-Crosshead speed

Studies showed contradictory results regarding the effects of crosshead speed on the SBS and TBS. For example, Sood **et al**., [85] indicated that crosshead speed variation between 0.5 and 10 mm/min did not have an influence on diametral tensile strength of a resin composite. However, another study reported statistically higher bond strengths for specimens loaded at 1.0 and 5.0 mm/min compared to 0.5 and 0.75 mm/min [86]. Regarding the effect of crosshead speed on µTBS, many studies have shown that it seems to be minimal [87, 88].

#### c- Gripping devices

Test specimens can be usually held in place by active or passive gripping approaches in order to apply a tensile load by aligning a specimen's bond line with its gripping surfaces. An active gripping approach includes gripping of the specimen to a gripping device using mechanical means or a fast-setting glue. However, the passive gripping method involves placement of the specimen in a testing device without mechanical gripping. The specimen gripping procedures require special test jigs, such as the Bencor multi-T gripping device and Ciucchi's jig where the specimens are glued to a flat gripping surface, thus resulting in possible malalignment [41]. Geraldeli's jig and modified Ciucchi's jig were used to enhance the specimen alignment [89]. A self-aligning, glueless, passive gripping device, named as Dirck's device was introduced recently [89]. The advantage of this device is that it is less sensitive to human errors than Geraldeli's jig, and results in a more uniform stress distribution at the dumbbell specimen adhesive layer than does the Geraldeli's device at the stick layer [32].

## 7 RECOMMENDATIONS TO IMPROVE THE VALIDITY OF BOND STRENGTH TESTING METHODS

1. When assessing bonding efficacy, researchers should consider improving standardization of the study variables of the laboratory bond strength tests following the guidelines of the International Organization for Standardization (*i.e*. ISO Technical Specification [No. 11405] on ‘Testing on adhesion to tooth structure’).
2. Standardized study variables in laboratory bond strength tests should be combined with standardized data from microleakage tests, gap evaluation tests, and bond durability tests for a better clinical correlation.
3. The validity of laboratory bond strength tests can be enhanced by the implementation of standardized protocols in the test method and combining dynamic fatigue test results.
4. New adhesive materials should be subjected to a combination of testing protocols to properly assess their bonding effectiveness.

## CONCLUSION

Bond strength testing methods for assessment of bonding effectiveness should be well- standardized since there are many factors that affect bond strength measurement, and some of these factors cannot be fully controlled which may lead to variations. Results of the current bond testing methods should be used to compare materials tested under the same laboratory settings, but they shouldn’t be used to make direct inferences on their clinical behaviour. Shear and micro-shear tests, result in non-uniform stress distribution, stress concentration at the substrate area, and predominantly tensile stresses rather than shear stresses. Micro-tensile bond tests provide many advantages over the shear tests, although these methods are technique sensitive and labour intensive. Currently, the challenge in adhesive dentistry is to make the adhesive-tooth interface more resistant to aging, thereby rendering the restorative treatment more predictable in terms of durability and clinical performance in the long run.

## CLINICAL SIGNIFICANCE

New adhesive materials should be subjected to a combination of testing protocols to properly assess their bonding effectiveness.

## Figures and Tables

**Fig. (6):**
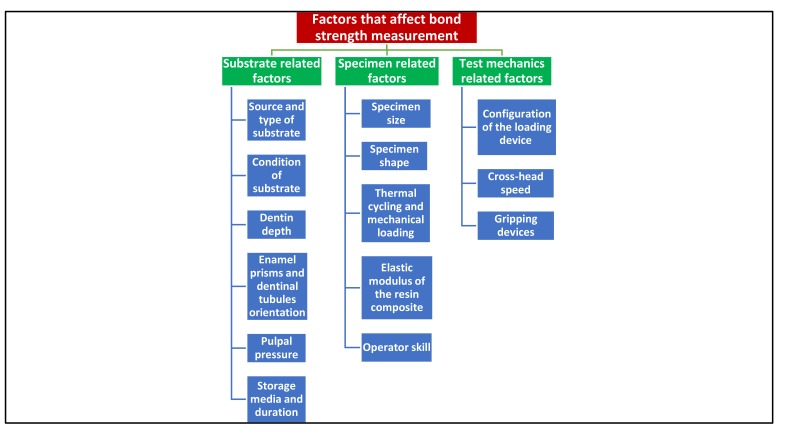
Factors that affect bond strength measurement.
